# Rheumatic disease patient decision-making about COVID-19 vaccination: a qualitative analysis

**DOI:** 10.1186/s41927-022-00307-6

**Published:** 2022-11-29

**Authors:** Yomei P. Shaw, Sara Hustek, Nina Nguyen, Makenzie Starlin, Kristin Wipfler, Beth I. Wallace, Kaleb Michaud

**Affiliations:** 1 FORWARD, The National Databank for Rheumatic Diseases, Wichita, KS USA; 2 University of Michigan Division of Rheumatology, Ann Arbor, MI USA; 3 University of Nebraska Medical Center, Omaha, NE USA; 4 Center for Clinical Management Research, Veterans Affairs Ann Arbor Healthcare System, Ann Arbor, MI USA

**Keywords:** COVID-19, Vaccines, Qualitative research, Medication adherence, Rheumatic diseases

## Abstract

**Background:**

Although patients with rheumatic and musculoskeletal diseases (RMDs) are at increased risk for adverse outcomes of COVID-19 illness compared to healthy controls, they also have lower rates of willingness to be vaccinated. Previous research has identified reasons for vaccine hesitancy among patients with RMDs (such as concerns about side effects and flares), but little is known about what these reasons mean in the context of patients’ lives, or how vaccine decision making is experienced from a patient perspective. Our objective was to describe decision-making about COVID-19 vaccination among RMD patients.

**Methods:**

Participants in a RMD registry were invited to complete monthly online surveys regarding COVID-19 vaccination from March-June 2021. We qualitatively analyzed comments from two open-ended survey questions reporting general experiences with vaccination and side effects. Comments were coded for attitudes towards COVID-19 vaccination, vaccine access, rheumatologic medication management around vaccination, and vaccine side effects. Themes were identified for the process and context of COVID-19 vaccine decisions, patient motivations for receiving or avoiding vaccination, and consistency of peri-vaccine medication management with current ACR guidelines.

**Results:**

We analyzed 710 comments from 537 respondents. Commenting respondents had a mean age of 64 years, were 87% female, 94% white, and 93% received/intended to receive ≥ 1 dose of a COVID-19 vaccine. Desire for protection and a return to normal routines motivated some commenters to get vaccinated, while concerns about vaccine side effects motivated others to delay or avoid vaccination. Several commenters reported disease flares following vaccination. Some commenters did not consult their providers about vaccination and failed to withhold immunomodulatory medications during vaccination, while others withheld medications more conservatively than recommended by current ACR guidelines, either on their own or directed by their provider.

**Conclusions:**

While most commenters were vaccine-accepting, challenges to COVID-19 vaccine uptake in the RMD population may include fears of side effects, including worsened RMD symptoms, and perceptions that vaccination is unnecessary. Addressing these concerns and beliefs may be critical for promoting vaccination in this population.

**Supplementary Information:**

The online version contains supplementary material available at 10.1186/s41927-022-00307-6.

## Background

Patients with rheumatic and musculoskeletal diseases (RMDs) accurately view themselves as particularly vulnerable to adverse outcomes of coronavirus disease 2019 (COVID-19) [1–5]. However, they are also less willing than healthy controls to receive COVID-19 vaccination [4, 6, 7]. A previous survey study of participants in FORWARD, the National Data Bank for Rheumatic Diseases found that reasons for vaccine hesitancy among RMD patients include concern about side effects, lack of trust in the science/government/pharmaceutical companies, lack of testing in patients with rheumatic diseases or other conditions, and fear of flares [8]. However, little is known about the significance of these reasons in the context of RMD patients’ lives or how they make decisions about COVID-19 vaccination.

The American College of Rheumatology (ACR) has consistently recommended COVID-19 vaccination for RMD patients [9–13]. Initial guidelines have been updated to recommend withholding doses and/or altering vaccine timing for patients taking disease-modifying anti-rheumatic drugs (DMARDs) [13]. Knowing how and why DMARD management and vaccine timing may differ from these recommendations may help clinicians ensure optimal COVID-19 protection for RMD patients.

In this study, our objective was to qualitatively examine important considerations and motivations for RMD patients making decisions about COVID-19 vaccination. We conducted a post hoc qualitative analysis of free text comments shared by FORWARD participants who had been surveyed about their attitudes towards and experiences with COVID-19 vaccine from March-June 2021.

## Methods

We conducted a post hoc thematic analysis of free-text comments from an online survey about COVID-19 vaccination informed by an interpretative phenomenological approach. Interpretative phenomenological analysis aims to elucidate participants’ lived experience of a phenomenon, while considering the influence of the social and historical context on how individuals make sense of and respond to situations [14–16]. This approach is suitable for exploring how patients with chronic conditions engage in a health-related decision [17–20]. Identification of themes related to peri-vaccination DMARD management was also informed by the ACR guidelines on COVID-19 vaccination [13].

### Study population

Study participants were enrollees in FORWARD, a United States-based registry of patients with RMDs [21]. The registry recruits patients through rheumatology providers as well as directly through its website. FORWARD thus includes participants with systemic autoimmune diseases, as well as those with non-systemic rheumatic conditions common in the general population of the United States. FORWARD participants complete questionnaires about their health status and medications bi-annually. From March-June 2021, FORWARD participants were invited to complete monthly online surveys about COVID-19 vaccination [8, 22].

### Data collection

Survey topics included COVID-19 vaccine intentions and decision-making, DMARD management peri-vaccination, and vaccine side effects (see Additional file 1). Two optional open-ended questions provided data for our analysis (see Additional file 2). The study was approved by the Institutional Review Board of Via Christi Hospitals Wichita, Inc (FWA00001005; IRB00001674). Written informed consent was obtained before patients completed surveys.

### Data analysis

The unit of analysis was each free text comment. Free-text data was reviewed by four authors (YPS, SH, NN, MS) who proposed a codebook for labeling the comments for relevancy to COVID-19 vaccination and topics of interest, including vaccine intentions, concerns, access, and peri-vaccine medication management. Three authors (SH, NN, and MS) coded the data using Microsoft Excel. Each comment was coded by two authors. Pairs of coders resolved discrepancies in coding by discussing until consensus was reached.

After excluding comments irrelevant to COVID-19 vaccination, thematic analysis was performed by SH, NN, MS and YPS. Team members carefully re-read the comments grouped by codes and took notes about patterns related to vaccine intentions, the patient decision-making process, motivations for vaccine decisions, and DMARD management peri-vaccination. Themes were drafted based on notes and refined through weekly group discussions and further review of comments and note taking.

## Results

**Fig. 1:**
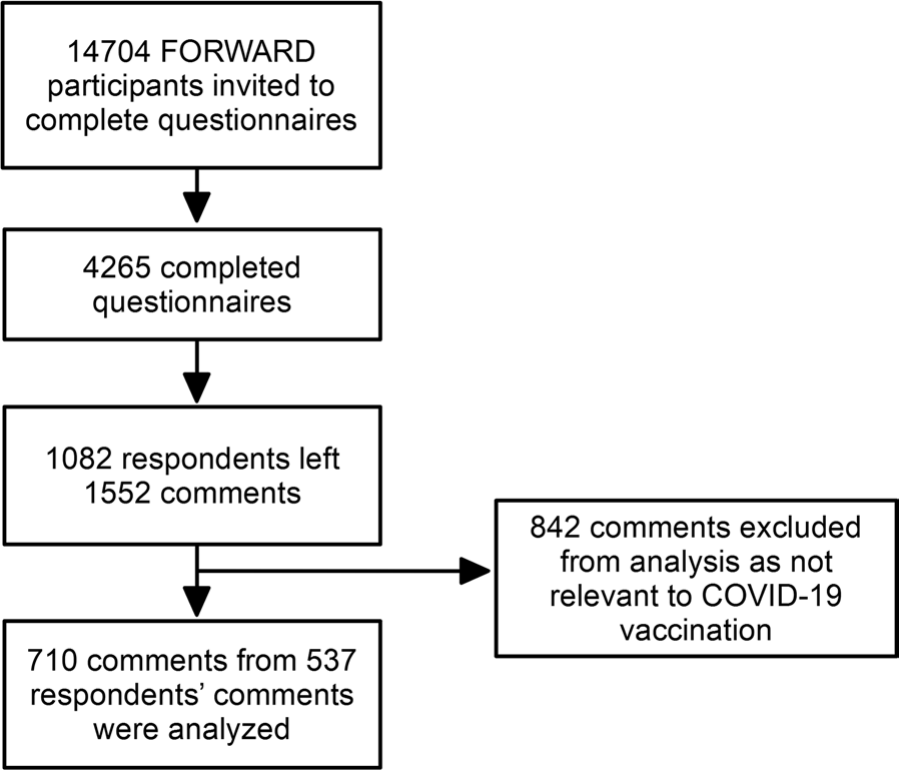
Study flowchart

Out of 14,704 participants invited, 4,265 completed ≥ 1 COVID-19 vaccine questionnaires; 1082 respondents left a total of 1552 free text comments. After excluding irrelevant comments, 710 comments from 537 commenters were analyzed (Fig. 1).

Table 1 shows the characteristics of these 537 commenters. Sixty-four percent were 65 or older, 87% were female, 94% were white, and 15% reported previous COVID-19 illness. Although the majority (93%) received/intended to receive at least one dose of a COVID-19 vaccine, concerns about vaccines were commonly expressed (83%) among both vaccinated and unvaccinated commenters. Concerns included side effects (71%) and the effects of vaccination on DMARD management and flares (20%).

**Table 1:** Characteristics of participants included in the analysis (n = 537)

Characteristic	Mean ± SD or Frequency (%)
Age (years)	64.4 ± 11.9
≥ 65 years old	288 (53.6)
Female	468 (87.2)
White	503 (93.6)
Education (years)	15.3 ± 2.2
Rural residence	116 (21.7)
United States resident	503 (93.7)
*Primary diagnosis*^a^	
Rheumatoid arthritis	291 (56.6)
Dupuytren’s	90 (17.5)
Osteoarthritis	43 (8.4)
Systemic lupus erythematosus	22 (4.3)
Fibromyalgia	24 (4.7)
Psoriatic arthritis	14 (2.7)
Ankylosing spondylitis	5 (1.0)
Other	25 (4.9)
*Comorbid conditions*^b^	
History of cancer	156 (33.3)
History of pulmonary disorder	251 (53.6)
History of heart disease	204 (43.6)
*Disease activity*	
Pain Visual Analog Score (0–10)	4.0 ± 2.8
Patient global Visual Analog Score (0–10)	3.5 ± 2.4
HAQ-II (0–3)^g^	0.7 ± 0.6
*Medications*^c^	
Any conventional synthetic DMARD^g^	219 (49.8)
Methotrexate	132 (30.0)
Leflunomide	29 (6.6)
Sulfasalazine	18 (4.1)
Mycophenolate	4 (0.9)
Cyclophosphamide	1 (0.2)
Any biologic DMARD^g^	174 (39.5)
Rituximab	17 (3.9)
Janus kinase inhibitor	18 (4.1)
Glucocorticoid	90 (20.5)
*COVID-19*^g^ *vaccine intentions*	
Not intending to get vaccinated/undecided	36 (6.7)
Vaccinated/intending to get vaccinated	531 (93.3)
*Peri-vaccine DMARD*^g^* management*	
No medication changes^d^	387 (77.6)
Medication changed at direction of physician^d^	72 (14.4)
Medication changed by patient^d^	57 (11.4)
*Vaccine decision factors/attitudes*	
COVID-19^g^ positive (currently or in the past)^e^	81 (15.1)
Frustrations with vaccine access	33 (6.1)
Reported COVID-19^g^ vaccine side effects^d^	411 (76.5)
Expressed any concerns about vaccine^f^	447 (83.2)
Concerns about side effects	384 (71.5)
Concerns about DMARD^g^ management/flares	105 (19.6)
Concerns about effectiveness	27 (5.0)
Mistrust/vaccine not needed	20 (3.7)

^a^Diagnosis was available for n = 514

^b^Comorbidity information was available for n = 468

^c^Medication information was available for n = 440

^d^Among those vaccinated (n = 499). Medication could be changed by patient, physician, both, or neither

^e^Ever reported COVID-19 diagnosis to FORWARD on previous questionnaires

^f^It was possible for individual commenters to be concerned about multiple aspects of the COVID-19 vaccines, so that the below percentages do not add up to 83.2%

^g^COVID-19: Coronavirus disease of 2019; DMARD: disease-modifying anti-rheumatic drug; HAQ: Health Assessment Questionnaire

Thematic analysis yielded 15 subthemes organized into four main themes: process and context of vaccine decision-making, motivations for receiving a COVID-19 vaccine, motivations for avoiding vaccination, and peri-vaccine DMARD management. Table 2 presents the themes, subthemes, and illustrative quotes.

**Table 2:** Themes, subthemes, and illustrative quotes

Theme	Subtheme	Illustrative quote
Process and context of vaccine decision making	Ongoing process of weighing risks and benefits of vaccination	We dont have clear access to the vaccines other than Astra Zeneca, and aside from Duputryns in my left hand, given my history of DVT and high BP, combined with Australia's current May 2021 control of COVID19 locally, I dont like the odds of a vaccine and potential side effects vs staying away from COVID locally
	Involvement of health care provider(s) in decisions	I discussed stopping my medications after the first shot with my doctor. She said it was my choice but it would help me build a somewhat stronger immunity to Covid if I did. So, I stopped taking my methotrexate and Celebrex. Today, almost 5 weeks later, I restarted those medications. In two days, it will be two weeks after my second shot. The pain from my arthritis was becoming pretty intense
	Vaccine access	I'm somewhat afraid of the mRNA-based vaccines (Pfizer & Moderna), but realize that the risks of harm from Covid-19 are much greater. So I will not delay, although the Johnson&Johnson vaccine would be preferred, and the AstraZeneca next (both not available here till April at the earliest). I'll just brace for the side effects, especially after the second jab, which is said to be more problematic
	Previous or current COVID-19 illness	I had COVID in January of this year. I received an antibody infusion (Bamlanivimab) and cannot receive the vaccine until 90 days have passed. I will be able to receive the vaccine after April 11, 2021
Motivations for receiving a COVID-19 vaccine	Desire for safety	How to get vaccinated! I also work with children. I really need to get vaccinated. It seems that having RA and taking medication that suppress my immune system, does not matter in order to get the vaccine
	Desire to return to normal routines	I was unable to continue Orencia infusions when the pandemic started. Because I have been vaccinated, I will be able to start infusions again soon. The replacement medications, sulfasalazine, hydroxychloroquine and prednisone have not worked well enough for my RA pain
	External pressures to vaccinate	The main reason for getting the vaccine was to get on with life, which it was appearing to be much more difficult if you didn't comply with getting one, not because I felt I needed it to be safe
Motivations for avoiding COVID-19 vaccination	Concerns about side effects	Very nervous about having my vaccine shots with having Lupus. the aftereffects of the second shot can be severe. waiting for the J &J shot. they are supposed to be safer
	Concerns about DMARD management/flares	Still not positive I will get vaccine. I do not want to be off RA meds for 2 weeks before and after each shot
	Perception of low risk	There are very effective treatments and therefore no "vaccine" needed. The survival rate is very high for most people. The VAERs system is recording many, many health issues. No thanks to this experimental biological agent
	Doubts about vaccine effectiveness	As time progresses, I am receiving more and more diagnoses. I started with Dupuytren's Contracture. I now have Dupuytren's, Osteoarthritis in both hips, both wrists and lower spine. I just don't want to add side effects from a vaccine to make my life even more miserable, especially when these vaccines don't stop COVID-19, they just reduce the severity of it
	Mistrust	I don't trust Biden or Fauci, will never get the vaccine unless physically coerced
Peri-vaccine DMARD management	Need for guidance on DMARD management	Concerned over how to hold medications during the vaccine cycle
	Failure to withhold DMARDs	I was vaccinated and still got COVID. I think that I was on methotrexate when vaccinated may have had something to do with me getting sick
	Withheld DMARDs more conservatively than recommended by current ACR guidelines	I was advised to skip my weekly Enbrel injections until 2 weeks after I finish my Covid vaccines

COVID-19: Coronavirus disease 2019; DMARD: Disease-modifying anti-rheumatic drug; ACR: American College of Rheumatology; DVT: Deep Venous Thrombosis; BP: Blood pressure; mRNA: Messenger ribonucleic acid; RA: Rheumatoid Arthritis; VAERS: Vaccine Adverse Event Reporting System

### Process and context of vaccine decision making

Commenters were categorized into three main groups based on COVID-19 vaccination intentions: (1) intending to get or already vaccinated, (2) unsure about vaccination, and (3) not intending to get vaccinated. Many in group 1 expressed eagerness and optimism about the protection they hoped the vaccine would provide or gratitude and relief about getting vaccinated. Some even positively interpreted perceived side effects as a sign the vaccine was working:

I had a reaction after both vaccine injections which made me very happy! Yippee because it means I mounted an immune response.

In contrast, commenters in group 3 resisted vaccination because they believed vaccines were unsafe or unnecessary, or they wished to avoid unwanted effects on their RMD or other health issues.

Attitudes towards vaccination in group 2, the undecided commenters, were more mixed. Some commenters strongly preferred to avoid vaccination but remained willing to be vaccinated if required. Others were interested in vaccination but wanted input from a health care provider first. Others wanted to wait for more evidence on how the vaccine might affect their health condition(s):

I need it to be tested and studied in folks with fibromyalgia and orthostasis and if it can be used safely used for us with these conditions, then I might get the vaccine, just not until then […]

#### Subtheme: Ongoing process of weighing risks and benefits

Decision-making about vaccination often involved monitoring factors that evolved throughout the pandemic, such as the local level of COVID-19 transmission, availability of vaccines, and their reported risks. The decision process continued even after commenters were vaccinated. One commenter’s decision was influenced by their experience of side effects after their first vaccine dose:

I got the petechiae about a week after the shot. Because of that, I decided against getting the 2nd dose.

#### Subtheme: Involvement of health care providers in vaccination decisions

Some commenters made vaccine-related decisions without consulting providers or received provider advice only after their first dose. Others actively sought rheumatologist advice about vaccine choice, timing, and DMARD management. Because vaccine-related knowledge and recommendations were rapidly evolving, instructions from rheumatologists also changed over time:

My rheumatologist for first vaccine told me to wait three days after shot and then resume mtx but no changes in Arava. […] Between first and second vaccine shot, rh academy changed advice and so I was to wait 7 days after vaccine shot.

#### Subtheme: Vaccine access

Vaccine access was an important contextual factor shaping commenters’ choices. Throughout March-June 2021, when comments were submitted, COVID-19 vaccine supplies were limited, and availability depended on the commenter’s location and date of response. While some commenters had early access to vaccines, e.g. through work, many others reported frustration with the difficulty of obtaining vaccination and criticized local policies affecting vaccine access. For example, one commenter complained about Canada’s policy of delaying second vaccine doses until most people received a first dose. Commenters were also frustrated that people with RMDs were not prioritized for vaccination along with other high-risk groups.

Commenters preferred certain vaccines for safety or convenience reasons. Some even delayed vaccination due to dissatisfaction with available options in their region:

Very nervous about having my vaccine shots with having Lupus. the aftereffects [sic] of the second shot can be severe. waiting for the J &J shot. they are supposed to be safer.

#### Subtheme: Previous or current COVID-19 illness

Some commenters who contracted COVID-19 were advised to wait before receiving a vaccine:

Was diagnosed with COVID-19 in March, spent 10 days in the hospital, doctor said to wait 6 months before trying to get the vaccine.

Others were fearful of the vaccine due to their experiences with COVID-19 illness:

I had a bad cough, fever, and could not sleep in my bed because I couldn't breathe when I laid down, so I slept in my recliner. I coughed for nearly 6 weeks […] I am doing very well now and do not want to risk the virus shot.

### Motivations for receiving a COVID-19 vaccine

#### Subtheme: Desire for safety

Many commenters sought vaccination to protect themselves from COVID-19. Some perceived themselves as especially vulnerable due to their health conditions:

In addition to RA I am also immuno-compromised due to subtotal colectomy, chronic kidney disease, fibromyalgia, anemic, a [sic] several other conditions. I have been fearful of coming down with the Covid virus.

Other commenters voiced concern about exposure to COVID-19 at work: “I am 67 and working full time as a Nurse Leader in a medical center and see COVID patients occasionally in Perinatal.”

#### Subtheme: Desire to return to normal routines

During the pandemic, commenters paused treatment routines and daily activities to reduce risk of COVID-19 illness and severe outcomes. Vaccination was seen as the pathway to safely resuming needed treatments for RMDs and valued activities:

I am hoping that after my second vaccine I can go back to the gym.

#### Subtheme: External pressures to vaccinate

External pressures were also important motivators, especially for those uncertain about vaccination. Some commenters were willing to consider vaccination if it was required/mandated, or if requested by family or friends:

If there comes a point where we all must produce vaccine passports in order to travel internationally, then I will get the vaccine but until then, I don't plan on getting it.

If I am forced to get one because of other people's fears, ie. wouldn't be able to see family members if I don't get one […] I will consider it […]

### Motivations for avoiding COVID-19 vaccination

#### Subtheme: Concerns about side effects

Vaccine side effects were an important concern for many commenters, even those planning to be vaccinated. Commenters were afraid of serious side effects (e.g. blood clots and severe allergic reactions) as well as long-term consequences:

I’m afraid of the long term effects that can be serious to us and what harm it will do to our young children. I wish I had not gotten it.

Others were afraid the vaccine would weaken their immune system: “not interested in getting a vaccine thay [sic] may affect my body's immune system.” Some commenters were not willing to risk facing vaccine side effects in addition to existing health problems:

As time progresses, I am receiving more and more diagnoses. […] I just don't want to add side effects from a vaccine to make my life even more miserable, especially when these vaccines don't stop COVID-19, they just reduce the severity of it.

Even those who got vaccinated were apprehensive about their second dose, which was believed to have greater side effects than the first dose. Some concerns were based on other vaccine experiences:

10 years ago I had a very bad reaction to a pneumonia shot. I have mailed a lot of information to my rheumatologist and internist and am waiting on their advice.

#### Subtheme: Concerns about DMARD management/flares

Concerns about DMARD management and RMD flares were important for many commenters, regardless of vaccination intention. Some commenters did not want to interrupt their DMARD treatment for vaccination:

My rheumatologist said I would have to be off the orencia infusion for 3 months to complete the vaccination process. I'm just not willing to do that at this point. I am finally feeling a little better.

In this case, the rheumatologist’s recommendation was more conservative than ACR guidelines and resulted in the commenter’s decision against vaccination. Commenters also wanted to avoid flares to maintain social and employment roles:

I feel it is more important for me to be strong enough to care for her [mother-in-law] than to risk having a flare [from vaccination] that could debilitate me for weeks.

Some commenters who chose to get vaccinated also reported negative experiences with post-vaccination flares. Some directly attributed their flare symptoms to vaccination: “I had the worst flare up that I've had in years following the vaccine.” Others were concerned about vaccination triggering disease progression or worsening:

Yesterday was the two-week point after my second shot, yet I still felt chills and debilitating fatigue. I hope this is temporary and not because the vaccination caused my fibromyalgia symptoms to re-emerge.

In other cases, commenters felt that withholding DMARDs during vaccination might have worsened RMD symptoms:

Definitely missed the dose of my Cimzia. It was delayed by 3 weeks due to vaccinations. Also had my epidural injections delayed by 5 weeks due to the vaccinations. Very hard to move around without pain and sciatic involvement.

A few commenters reported severe post-vaccination flares that impacted daily functioning:

As with the first dose, I had one day of overall body aches that felt like an RA flare. But then a couple of days later, the missing Xeljanx hit me HARD. Everything hurt, I had little energy, and I was nauseated (a fairly typical RA flare presentation for me, but far worse). I even took a half-day off from work, which is rare for me […] It was a horrid experience, and I'll be hesitant to take similar advice again.

#### Subtheme: Perception of low risk

The perception that COVID-19 posed little risk contributed to some commenters viewing vaccination as unnecessary. Some believed they were unlikely to become infected because of precautions they were taking, or because their immune system would protect them:

During the pandemic last February I traveled throughout SE Asia. I was in rooms with thousands of people in Seoul airport, in many crowds of people in Vietnam and Burma […] all with no politically correct masks. Having a healthy immune system via a plant-based lifestyle is more important than subjecting your body to experimental drugs.

Some commenters thought the risk of COVID-19 illness was exaggerated (“The survival rate is very high for most people”) or trusted they could be successfully treated if infected (“I have 2 doctors with 100 percent success rate treating COVID. No need for an experimental biological agent”).

#### Subtheme: Doubts about vaccine effectiveness

Doubts about vaccine effectiveness caused some commenters to question whether vaccines were worthwhile. Some believed vaccines were ineffective for preventing COVID-19 illness: “don’t want to risk and add more to my problems especially since the vaccine DOES not prevent or cure Covid.” One commenter wondered whether vaccines benefited patients with previous COVID-19 illness:

I have heard nothing from doctors or scientists to show why people who had Covid should get a vaccine. Some say that the antibodies only last in the body for a few months, so why does the vaccine last longer? What evidence shows the length of time the vaccine lasts?

Many commenters also wondered whether RMD treatments would reduce vaccine effectiveness, although this concern typically did not dissuade commenters from vaccination. A few commenters were worried by negative antibody test results following vaccination: “Others I know on meds for autoimmune diseases are also testing negative for antibodies. What a false sense of protection since Feb./March.”

#### Subtheme: Mistrust

Mistrust reinforced commenters’ fears about potential side effects of COVID-19 vaccines. Misconceptions about the nature of the vaccines and how they work, as well as the vaccine testing and approval process, contributed to commenters’ suspicions. Some cited a lack of trust in political leaders, health authorities, or pharmaceutical companies:

I don't mind vaccines in general, but this particular vaccine I don't trust. You would expect mild side effects from vaccines like the flu. Not the serious side effects of the COVID vaccine, or even deaths. Don't trust the drug companies for this specific vaccine.

Commenters mistrusted mRNA vaccines, which were perceived as a new and unfamiliar technology: “There is zero short or long term data on manipulating the immune system to generate a non human protein. We have no idea what will happen to the masses who took the mRNA or DNA injections.” Commenters also worried about becoming a ‘guinea pig’ by taking vaccines perceived as ‘experimental’ and inadequately tested or reviewed:

I would never jeopardize my health and well-being by talking [sic] the experimental gene therapy shot they have mislabeled as a vaccine.

They went through FDA processes to approval to [sic] fast for my liking.

### Peri-vaccine DMARD management

#### Subtheme: Need for guidance on DMARD management

Commenters wanted to know whether DMARDs could reduce vaccine effectiveness, and how to manage DMARDS peri-vaccination. They often sought input from rheumatologists or other providers, such as infusion center staff and pharmacists. Commenters were sometimes confused about DMARD management peri-vaccination, either due to inadequate information or because the advice they received conflicted with how they usually managed DMARDs around other vaccinations:

I’m a little confused about the timing of receiving a Covid vaccination between Remicade infusions. My rheumatologist’s office gave me the impression I could get the vaccination any time but in the past I was told to time vaccinations half way between infusions so I’m waiting until then.

#### Subtheme: Failure to withhold DMARDs

Most DMARD management practices discussed by commenters were consistent with current ACR guidelines. However, in some cases, failure to withhold DMARDs or to withhold them for long enough may have reduced vaccine immunogenicity. For example, one commenter suspected that taking methotrexate during vaccination contributed to inadequate vaccine response:

I was vaccinated and still got COVID. I think that I was on methotrexate when vaccinated may have had something to do with me getting sick.

#### Subtheme: Withheld DMARDs more conservatively than recommended by current ACR guidelines

Others withheld DMARDs more conservatively than recommended by ACR guidelines. This was sometimes in addition to having stopped DMARDs due to concerns about immunosuppression as a risk factor for severe COVID-19 illness:

I have been compliant with all my RA meds for over 20 years, but as of December 2020 I stopped taking my Enbrel because my white blood cell count was very low…I was afraid if I got Covid I would not be able to fight it. I had no flare so I stayed off my meds (enbrel/methotrexate) until I received my vaccine.

Prolonged periods without regular DMARD use sometimes led to RMD symptoms worsening. For example, one commenter withheld both methotrexate and adalimumab:

[…] both [my medications] are on hold until two weeks after my second vaccine. I most definitely see a pain increase because of this, but feel the vaccine is quite important.

## Discussion

Our qualitative analysis highlights how various factors affect perceptions of vaccine risks and benefits among RMD patients. Commenters made decisions about COVID-19 vaccination through an ongoing process of weighing need for vaccination (risks of COVID-19, perceived vaccine effectiveness) against vaccine-related concerns (side effects and potential health impact, especially with respect to their RMD). This is consistent with the Necessity Concerns Framework (NCF), which posits that beliefs about medication necessity and concerns about adverse effects influence adherence behavior [23, 24]. The NCF was previously used to conceptualize COVID-19 vaccine willingness in young adults [25]. While most commenters were open to vaccination, many reluctant to get vaccinated had concerns about side effects and the potential for RMD flares, which they felt outweighed vaccine benefits. As mentioned in the results Sects. 3.3.1 (subtheme: concerns about side effects) and 3.3.2 (subtheme: concerns about DMARD management/flares), we found that even patients who intend to get vaccinated often have concerns about COVID-19 vaccines. It is important for rheumatologists to address patient concerns about COVID-19 vaccination, since patients must be willing to undergo periodic boosters in order to maintain protection against COVID-19. Patients whose concerns outweigh their perception of the benefits may ultimately decide not to get vaccinated for COVID-19 in the future, even if they have been willing to get vaccinated in the past. Rheumatology providers can help facilitate patient decision making about vaccines by providing information to promote more accurate perceptions of the risks and benefits of COVID-19 vaccination. Attitudes towards COVID-19 vaccines can change over time [26], and provider endorsement may increase vaccine uptake among the reluctant [27].

We identified motivations for resisting COVID-19 vaccines consistent with those previously reported [6, 7, 27, 28]. RMD patients may fear the vaccine’s impact on their health or mistrust those promoting vaccination. They may also doubt the vaccine’s effectiveness or prefer non-medical alternatives. Patients may also need time to consider vaccination, as they may be unconvinced by current safety data or overwhelmed while navigating multiple information sources. Some commenters had negative attitudes shaped by misinformation, as described in previous qualitative research. Reported examples of misinformation included rumors about minorities being targeted as ‘guinea pigs’, the idea that COVID-19 and/or vaccines were part of a conspiracy to control the population, and the belief that vaccines cause COVID-19 [29–31].

Commenters reported experiencing RMD flares following COVID-19 vaccination; these were attributed to withholding DMARDs, to direct effects of vaccination, or to a combination of both. Surveys of RMD patients found that 3–15% experience flares following COVID-19 vaccination, but most do not limit daily functioning [32–36].

As mentioned in the results Sect. 3.1.2 (subtheme: involvement of health care providers in vaccination decisions), patients sometimes looked to their rheumatologists and other health care providers for advice regarding COVID-19 vaccination, and it appears that a provider’s recommendation for vaccination or lack thereof was influential on patients’ decisions about vaccination. In a previous quantitative analysis of our survey responses, among unvaccinated patients, approximately 10% reported that lack of recommendation from the rheumatologist contributed to their reluctance to be vaccinated [8]. Among the cohort analyzed in this study, 140/238 (58.8%) reported that their rheumatologist recommended COVID-19 vaccination. However, commenters did not always seek provider input on coordinating vaccines with DMARDs. Commenters reported both failure to withhold and unnecessary withholding of DMARDs which potentially worsened RMD symptoms. Changing recommendations about DMARD management during our data collection period likely contributed to these experiences. Previous research supports our findings. A survey of vaccinated RMD patients found 18% of participants did not communicate with their provider about vaccination, and 71% did not withhold DMARDs peri-vaccination [36]. The prevalence of pandemic-period DMARD nonadherence is between 7.5 and 14.8% [37, 38]. To maximize vaccine effectiveness, it is important for rheumatologists to communicate with patients about coordination of DMARDs peri-vaccination. Patients want reassurance from health care providers about vaccine effectiveness and safety, and guidance about DMARD management.

The strengths of our study were our opportunity to collect comments from a large population of survey participants (4265) and that comments captured diverse attitudes towards vaccination. Our study also has limitations related to our data source. Commenters were all internet users, mostly elderly, white residents of the United States, and findings may not reflect attitudes of RMD patients with other sociodemographic characteristics. The majority of patients included in the analysis (56%) had rheumatoid arthritis, and many had non-systemic rheumatologic conditions. These characteristics of our study cohort limit the transferability of our findings to patients with other systemic autoimmune diseases, minorities, and younger patients. Future studies are needed to explore the attitudes of other RMD patient populations, including those in other parts of the world. Our results may also be affected by self-selection bias, since commenters may differ in important ways from those who did not respond or share comments. Since this was a post-hoc analysis of survey data, we did not have the opportunity to design a sampling strategy to focus on perspectives of subgroups of interest, such as patients with specific rheumatic diseases or the vaccine hesitant. The post-hoc nature of this analysis also precluded designing open-ended questions to capture perspectives in greater depth and with more consistency across respondents.

## Conclusions

Our qualitative analysis described RMD patient perspectives on COVID-19 vaccination, including the decision-making process, factors that encourage/discourage vaccination, and peri-vaccine DMARD management. Patients who are reluctant to vaccinate may perceive the risks of vaccination to be greater than the benefits. RMD flares are an important concern for patients both when deciding whether to receive vaccination as well as when evaluating their experience post-vaccination. There is a need for rheumatology providers to address concerns about COVID-19 vaccination, and to support patients in managing subsequent flares. Providing patients with accurate information about the risks and benefits of vaccines may enable the vaccine-hesitant to reevaluate their perceptions and make informed decisions about vaccination.

## Supplementary Information

**Additional file 1**. COVID-19 vaccine questionnaire. Questionnaire about COVID-19 vaccination that was administered to study participants online.

**Additional file 2**. Open ended survey questions. Free text responses to these open ended survey questions provided data for our analysis.

## Data Availability

The datasets used and/or analyzed during the current study are available from the corresponding author on reasonable request.

## Abbreviations

RMD: Rheumatic and musculoskeletal diseases
COVID-19: Coronavirus disease 2019
ACR: American College of Rheumatology
DMARD: Disease-modifying anti-rheumatic drugs
NCF: Necessity Concerns Framework
