# Supplementary material for: Rheumatic disease patient decision-making about COVID-19 vaccination: a qualitative analysis
Source: BMC Rheumatol. 2022 Nov 29;6:76. doi: 10.1186/s41927-022-00307-6 (PMC9706890; doi:10.1186/s41927-022-00307-6)
Supplement: Supplementary file 1 — Additional file 1. COVID-19 vaccine questionnaire. Questionnaire about COVID-19 vaccination that was administered to study participants online. [file 41927_2022_307_MOESM1_ESM.docx]

**Page 1: COVID-19 Vaccine**

*Page description: The answer to these questions are vital to guide public health decisions for the rheumatic disease community.*

In the past month, have you tested positive for COVID-19, or has a medical professional told you that you have COVID-19? (No / Yes)

Are **both** of the following statements true?

- I filled out the April COVID-19 questionnaire
- I received all of my COVID-19 vaccine shots before April

[If yes: skip to Page 2]

[If no:]

Has your rheumatologist/physician suggested you receive a COVID-19 vaccine?

(No / Yes)

[If yes:]

Which vaccine did you receive?

- Pfizer
- Moderna
- AstraZeneca
- Johnson & Johnson
- Don’t know, but it was only one dose
- Don’t know, but there were two doses
- Don’t know, but I’ve had one of two doses

[If 2 shots, or if identified one of the 2 dose vaccines:]

Did you receive both shots?

- - Yes🡪 How much time was there between your first and second shot?
    - 3 weeks
    - 4 weeks
    - 5 weeks
    - 6+ weeks
  - No, not yet
  - Don’t know

Did you have side effects with any shot of the vaccine you received?

No

Yes🡪 What were the side effects?

- Arm soreness
- Fever
- Muscle aches
- Fatigue
- Sleepiness
- Other________________

Did you or your doctor make any changes to your medication because of the vaccine? (This may include stopping or delaying a medication or infusion appointment.) (No / Yes )

[If no to vaccine:]

Have you had the opportunity to get the vaccine? (No / Yes)

Do you plan on getting the vaccine?

- No [go to “Why have you not…?”]
- Yes

Why have you not gotten/do you not plan to get the vaccine?

- Concerned about side effects
- Concerned it will cause a flare of my condition
- I don’t know about interactions with disease or medications
- I don’t know if it’s been tested on people with my condition or medications
- I don’t think I need it
- I don’t like the vaccines in general
- I don’t trust the science / review process / government / drug companies
- My doctor recommended against it
- My doctor has not recommended it
- Another doctor recommended against it
- Other _______________

**Page 2: PAS-II (named Your current health in the questionnaire)**

*Page description: The answers to the questions below give us a snapshot of how our participants feel at different stages of the vaccination process.*

Are you able to. . .

|  | **Without any difficulty** | **With some difficulty** | **With much difficulty** | **Unable to do** |
| --- | --- | --- | --- | --- |
| Get on and off the toilet? |  |  |  |  |
| Open car doors? |  |  |  |  |
| Walk outdoors on flat ground? |  |  |  |  |
| Stand up from a straight chair? |  |  |  |  |
| Wait in a line for 15 minutes? |  |  |  |  |
| Reach and get down a 5 pound object (such as a bag of sugar) from just above your head? |  |  |  |  |
| Go up two or more flights of stairs? |  |  |  |  |
| Do outside work (such as yard work)? |  |  |  |  |
| Lift heavy objects? |  |  |  |  |
| Move heavy objects? |  |  |  |  |

VAS: Considering all the ways that your illness affects you, rate how you are doing on the following scale.

VAS: We are also interested in learning whether or not you are affected by pain because of your illness. How much pain have you had because of your illness this past week?

Is there anything else you would like us to know? If so, tell us below:

___________________________________________________________
