# Supplementary material for: Rheumatic disease patient decision-making about COVID-19 vaccination: a qualitative analysis
Source: BMC Rheumatol. 2022 Nov 29;6:76. doi: 10.1186/s41927-022-00307-6 (PMC9706890; doi:10.1186/s41927-022-00307-6)

**Additional File 2.** Open-ended survey questions. #If participants selected ‘Other’, a free text field appeared allowing them to describe their side effect.


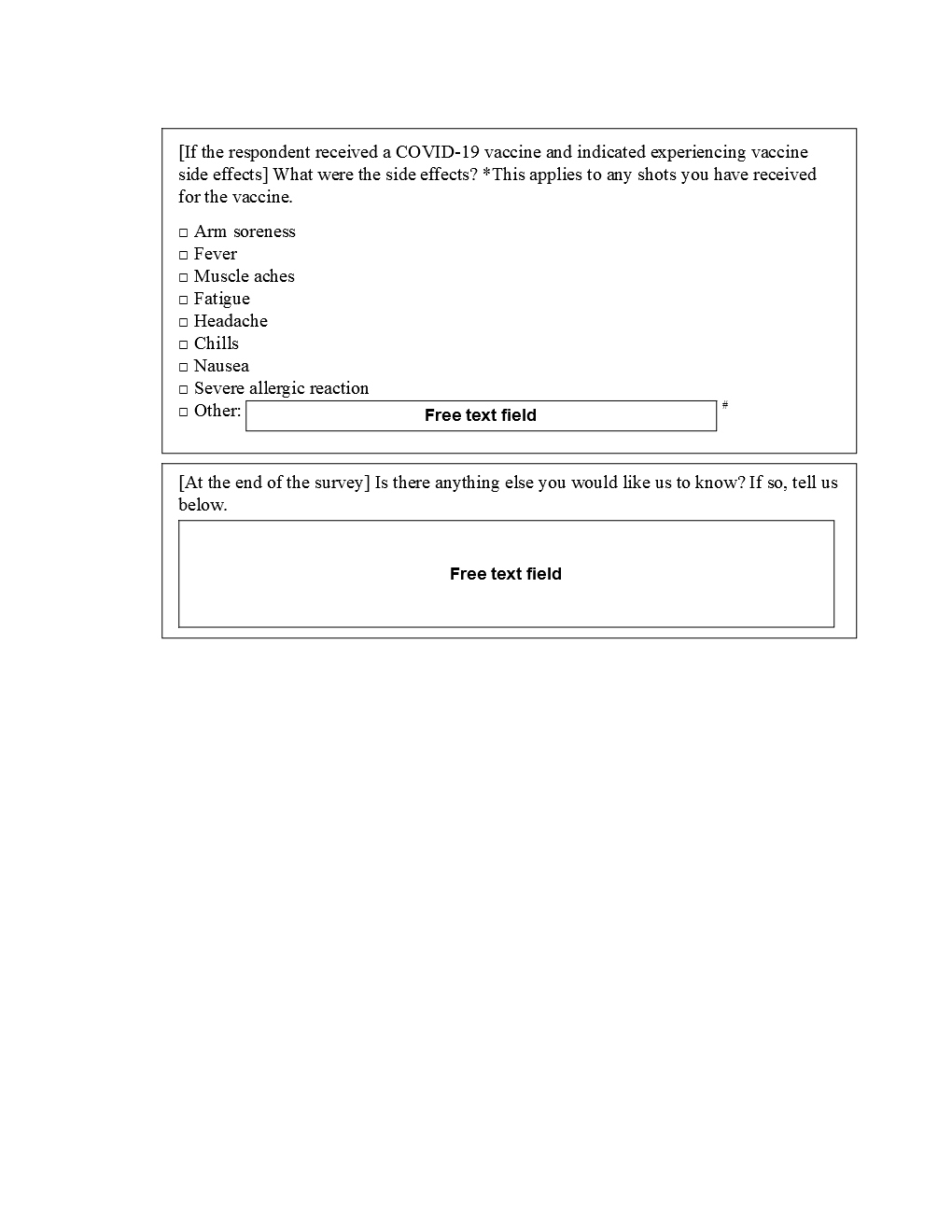

Supplement: Supplementary file 2 — Additional file 2. Open ended survey questions. Free text responses to these open ended survey questions provided data for our analysis. [file 41927_2022_307_MOESM2_ESM.docx]
